# Ca^2+^-dependent modulation of voltage-gated myocyte sodium channels

**DOI:** 10.1042/BST20200604

**Published:** 2021-10-13

**Authors:** Samantha C. Salvage, Zaki F. Habib, Hugh R. Matthews, Antony P. Jackson, Christopher L.-H. Huang

**Affiliations:** 1 Department of Biochemistry, University of Cambridge, Cambridge, U.K.; 2 Physiological Laboratory, University of Cambridge, Cambridge, U.K.

**Keywords:** C-terminal domain, ca^2+^, cardiac arrhythmia, cardiomyocytes, skeletal myocytes, sodium channels

## Abstract

Voltage-dependent Na^+^ channel activation underlies action potential generation fundamental to cellular excitability. In skeletal and cardiac muscle this triggers contraction via ryanodine-receptor (RyR)-mediated sarcoplasmic reticular (SR) Ca^2+^ release. We here review potential feedback actions of intracellular [Ca^2+^] ([Ca^2+^]_i_) on Na^+^ channel activity, surveying their structural, genetic and cellular and functional implications, translating these to their possible clinical importance. In addition to phosphorylation sites, both Nav1.4 and Nav1.5 possess potentially regulatory binding sites for Ca^2+^ and/or the Ca^2+-^sensor calmodulin in their inactivating III–IV linker and C-terminal domains (CTD), where mutations are associated with a range of skeletal and cardiac muscle diseases. We summarize *in vitro* cell-attached patch clamp studies reporting correspondingly diverse, direct and indirect, Ca^2+^ effects upon maximal Nav1.4 and Nav1.5 currents (*I*_max_) and their half-maximal voltages (*V*_1/2_) characterizing channel gating, in cellular expression systems and isolated myocytes. Interventions increasing cytoplasmic [Ca^2+^]_i_ down-regulated *I*_max_ leaving *V*_1/2_ constant in native loose patch clamped, wild-type murine skeletal and cardiac myocytes. They correspondingly reduced action potential upstroke rates and conduction velocities, causing pro-arrhythmic effects in intact perfused hearts. Genetically modified murine *RyR2*-P2328S hearts modelling catecholaminergic polymorphic ventricular tachycardia (CPVT), recapitulated clinical ventricular and atrial pro-arrhythmic phenotypes following catecholaminergic challenge. These accompanied reductions in action potential conduction velocities. The latter were reversed by flecainide at RyR-blocking concentrations specifically in *RyR2*-P2328S as opposed to wild-type hearts, suggesting a basis for its recent therapeutic application in CPVT. We finally explore the relevance of these mechanisms in further genetic paradigms for commoner metabolic and structural cardiac disease.

## Introduction

Transmembrane action potential initiation and propagation, mediated by surface membrane Na^+^ channel (Nav) proteins, is strategic to activation in excitable cells, of which skeletal and cardiac myocytes constitute important examples. The activation process feeds forward into a ryanodine receptor (RyR) mediated release of sarcoplasmic reticular (SR) store Ca^2+^. The consequent elevation of cytosolic Ca^2+^ concentration [Ca^2+^]_i_ is central to initiation of myocyte contraction. Ca^2+^ is additionally a strategic second messenger with signalling actions regulating protein activity through widespread cell types. This article addresses recent interest in possible Ca^2+^ feedback signalling on the Na^+^ channel itself, its possible physiological significance, and implications for human disease in skeletal and cardiac muscle. We relate the voltage sensing, and channel opening and inactivation processes in skeletal, Nav1.4 and cardiac Nav1.5 to their potential regulation at direct and indirect Ca^2+^ binding and phosphorylation sites. This includes its III–IV linker region and its interactions with its C-terminal domain, whose different regions are associated with widespread mutations related to skeletal and cardiac muscle disease. We examine *in vitro* studies in expression systems exploring for direct and indirect effects of Ca^2+^ on channel properties, then extend these to physiological studies in both skeletal and cardiac myocytes *in situ*, from experimental platforms using normal hearts, and those modelling genetic Ca^2+^ homeostatic disease, broadening these to genetic exemplars for more common human disease types.

## Membrane voltage-gated sodium channels underly excitable activity

**Figure 1.**
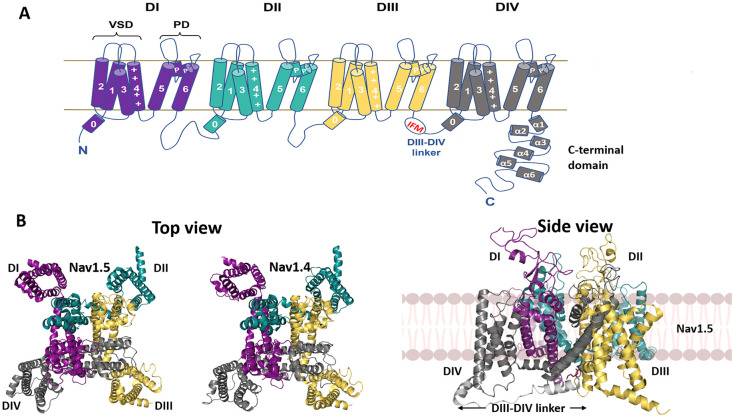
Structure of the Nav channel. (**A**) Key structural features of the Nav channel α-subunit. The four internally homologous domains, DI-IV, are colour-coded, with the S0 and transmembrane helices, S1–6, voltage-sensing domain (VSD), pore domain (PD), C-terminal domain and intracellular DIII-DIV linker region as indicated. (**B**) Cryo-EM structures of human Nav1.5 (PDB: 7dtc) and human Nav1.4 (PDB: 6agf) in top view and human Nav1.5 in side view. Domains colour-coded as in (**A**). The intracellular DIII-DIV linker is shown in the side view in light grey.

Voltage-gated sodium channels (Navs), expressed in excitable cells including neurons and skeletal and cardiac myocytes, initiate action potentials underlying electrical excitation and its propagation. Their principal α-subunits (Mwt ∼220–250 kDa) each include four homologous domains, DI-IV, each containing six transmembrane α-helices, S1–S6, following a S0 helix just preceding the S1 segment (Figure 1A). High-resolution structures obtained by cryo-electron microscopy (cryo-EM) of Nav1.4, Nav1.5 (Figure 1B) and other Nav subtypes demonstrate a highly conserved fourfold pseudosymmetric structure, with voltage sensing helices S1–S4 at the outer rim. Positively charged amino acid residues along one face of each S4 helix permit its outward rotation upon membrane depolarization. Transitions in the DI, DII and DIII S4 helices drive conformational changes in the tethered S5 and S6 helices forming the central pore region within each domain. These open the central, ion-selective pore, transitioning the channel from its resting, *closed* to an *open,* activated, state. The latter permits the inward, depolarizing, transmembrane Na^+^ fluxes driving cell excitation.

**Figure 2.**
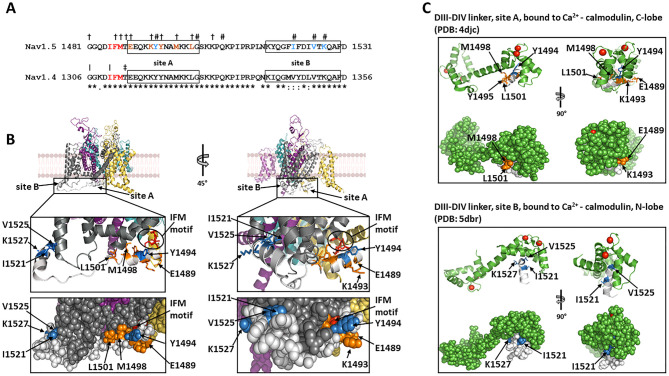
The intracellular DIII-DIV linker. (**A**) Sequence alignment of the DIII-DIV linkers from Nav1.5 and Nav1.4. Identical residues indicated by (*), conservative changes by (:) and semi-conservative changes by (.) below the sequence alignments. IFM motifs indicated in red. Site A and site B helices boxed. In the Nav1.5 sequence, examples of residues whose mutations are associated with Long QT syndrome (LQT3) indicated by (†) and with Brugada syndrome (BrS) by (#). LQT3 and BrS-associated residues implicated in binding of the DIII-DIV linker to the α-subunit and to Ca^2+^-calmodulin coloured orange and sky blue, respectively. In the Nav1.4 sequence, residues whose mutations are associated with myotonia indicated by (|) and with paramyotonia congenita (PMC) by (‡). (**B**) Expanded view of the Nav1.5 DIII-DIV linker (light grey), showing locations of the key residues coloured in (**A**), see text for details. (**C**). Binding of site A helix and site B helix to Ca^2+^-calmodulin C-lobe and N-lobe, respectively. Note the location of key site A and B residues coloured as in (**A**) and (**B**).

The slower outward movement of the DIV S4 helix then facilitates binding of a hydrophobic IFM (isoleucine, phenylalanine, and methionine) motif within the cytoplasmic III–IV linker (Figure 2A) to a hydrophobic pocket between domains III and IV (Figs. 1B and 2B) blocking the pore in the channel *inactivated* state, and restoring the resting membrane potential [1,2]. Protein purification inevitably requires cell lysis, dissipating the cell membrane potential: currently available Nav channel cryo-EM structures likely correspond to the inactivated state [2–6]. Indeed, these structures represent the IFM motif, as expected, engaged with an allosteric intracellular DIII site. In addition however, two separate, short α-helical regions of the DIII-DIV intracellular linker, site A and site B (equivalent to helix 0 of DIV: Figure 1A), make contacts with intracellular sites on DIV, probably further stabilizing the inactivated state (Figure 2A,B) [6]. However, if the engagement of the DIII-DIV linker and IFM motifs with these allosteric sites is indeed critical for promoting the inactivated state, then they must adopt different conformations in the resting and open states.

**Figure 3.**
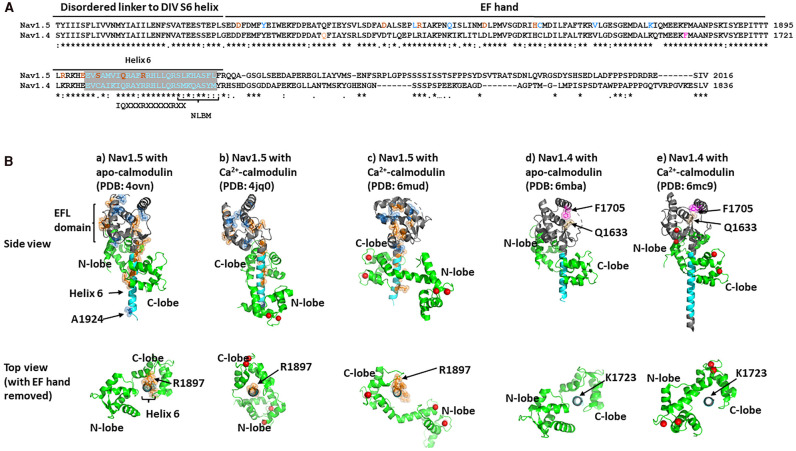
The Nav channel C-terminal domain (CTD). (**A**) Sequence alignment of CTDs from Nav1.5 and Nav1.4. Identical residues are indicated below the sequence alignment by (*), conservative changes by (:) and semi-conservative changes by (.). Locations of the linker region, EF hand and helix 6 highlighted. The extended region of helix 6 containing sequences implicated in apo- or Ca^2+^-calmodulin binding coloured cyan. Within this region, the consensus IQ-motif is indicated. In the Nav1.5 sequence, examples of LQT3 and BrS-associated residues coloured orange and sky blue, respectively. In the Nav1.4 sequence, myotonia and PMC-associated residues coloured tan and purple, respectively. (**B**) Comparative structures of CTDs from Nav1.5 (**a**–**c**) and Nav1.4 (**d**,**e**) with apo-calmodulin (**a**,**d**) or Ca^2+^-calmodulin (**b**,**c**,**e**), in side view and top view. To emphasize the variety of ways in which calmodulin can bind to helix 6, the EF hands have been removed from the top views and the orientation of each helix 6 structure has been arbitrarily standardized, with Nav1.5 residue R1897 and its Nav1.4 equivalent K1723 placed at 12 o'clock. In the Nav1.5 structures, LQT3 and BrS-associated residues highlighted as spheres and coloured orange and sky blue, respectively. In the Nav1.4 structures, myotonia and PMC-associated residues highlighted as spheres and coloured tan and purple, respectively. Ca^2+^ ions shown as red balls.

Nav channels also include a regulatory, globular, intracellular C-terminal domain (CTD), highly conserved amongst Nav subtypes (Figure 3A), connected to the DIV S6 helix via a flexible and disordered linker (Figure 1A). The CTD begins from amino acids 1599 in Nav1.4 and 1773 in Nav1.5, with a sequence of five α-helical regions fitting the consensus sequence for an EF-like hand (EFL) (Figure 3A,B) [7], with the latter part a fibroblast growth factor (FGF) homologous factor (FHF) binding site. It is followed by a sixth α-helical region (Figure 3A,B) and ends with a more disordered and less-well structurally characterized region containing short motifs likely controlling cytoskeletal binding and ubiquitination [8], including a Nedd4-like binding domain, PY motif domain and a syntrophin-anchoring PDZ binding motif (Figure 3A) [9].

**Figure 4.**
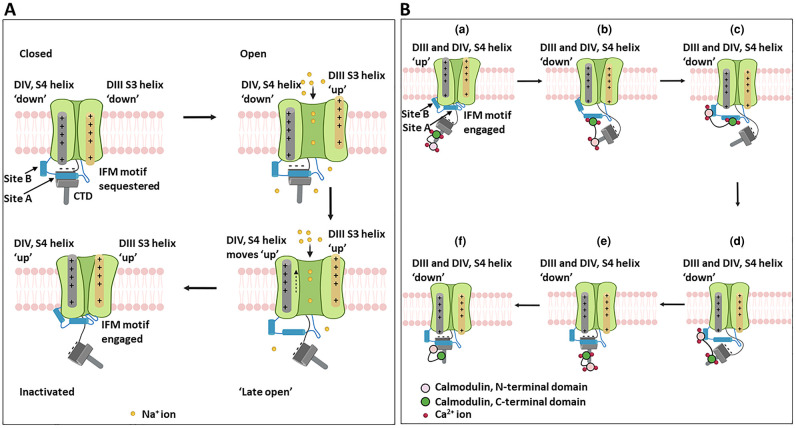
Proposed Nav channel conformational states during the action potential cycle. (**A**) Closed (resting), open (activated) and inactivated (refractory) states schematizing relationships between the activating DI-III (right side, orange), the inactivating DIV (left side, grey) voltage sensing domains, the CTD, and sites A and B of the intracellular III–IV linker. (**B**) Possible conformational relationships involving DIII-DIV linker and calmodulin during Nav1.5 recovery from inactivation. (**a**) Inactivated state, with IFM motif and DIII-DIV linker fully engaged with the α-subunit and the CTD dissociated from site A. Ca^2+^ levels are assumed to be elevated following opening of voltage-gated Ca^2+^ channels, so that Ca^2+^-calmodulin binds to helix 6 (PDB structure 4jq0). (**b**–**e**) Possible, sequential conformational changes occurring during the recovery from inactivation steps (see text for details). (**f**) Proposed Nav1.5 conformation after return to the closed (resting) state. Since Ca^2+^ levels are now low, apo-calmodulin binds to helix 6 (PDB structure 4ovn). Ca^2+^ shown as red balls.

NMR analysis of purified EFLs indicates the presence of a prominent cleft in the EFL, bounded by α-helices [10]. This cleft can complex with Site A of the DIII-DIV linker (Figure 2A,B). Modelling of dynamic interactions between the DIII-DIV linker and the CTD through the Nav channel cycle in mammalian Nav1.7 and cockroach NavPas channel structures [11] suggested that in the channel closed state, acidic residues on the CTD EFL domain form salt bridges with basic residues on the DIV S4 helix, whilst Site A of the DIII-DIV linker is held in the CTD EFL cleft. As a consequence, the IFM motif is physically constrained and prevented from prematurely engaging with the inactivated state [11,12]. Upward movement of the DIV S4 helix accompanying channel opening, disrupts these salt bridges. CTD dissociation from the DIII-DIV linker then frees the IFM motif permitting transition into the inactivated state (Figure 4A). Most of the cryo-EM structures thus do not show a resolved CTD [2–5]. This suggests that in the inactivated state, the CTD is free to adopt multiple conformations with respect to the bulk of the Nav channel, constrained only by its tethering to the S6 helix [11].

## Intracellular Ca^2+^ as potential Nav modulator

In skeletal and cardiac muscle, the RyR-mediated SR Ca^2+^ release following Nav1.4 or Nav1.5-mediated depolarization elevates bulk [Ca^2+^]_i_ from ∼100 nM to 1–10 µM causing contractile activation. In addition, recent reports implicated cytosolic Ca^2+^ in a *feedback* Nav modulation whether through Ca^2+^ by itself or following its binding to the modulator protein calmodulin [8,13]. The latter nM-low µM K_d_ Ca^2+^ sensor contains N- and C-lobes, each possessing two Ca^2+^-binding EF hands. In turn, Ca^2+^-free, apo-, calmodulin shows ‘closed’ and ‘semi-open’ states, while Ca^2+^ bound Ca^2+^-calmodulin shows ‘open’ and ‘semi-open’ states. EF hand helix orientations in the ‘open’ and ‘semi-open’ states expose a hydrophobic groove capable of binding distinct α-helical protein sequence motifs. [14–16].

Biophysical studies on isolated protein fragments demonstrate that Site A and Site B of the Nav DIII-DIV linker bind to the C- and N- lobes of Ca^2+^-calmodulin, respectively. But this interaction does not occur with the C- or N-lobes of apo-calmodulin (Figure 2A,C) [17,18]. As noted above, Site A can also bind the CTD EFL cleft. Interestingly, Nav1.5 DIII-DIV linker and CTD co-precipitation occurs in the presence of Ca^2+^-calmodulin, but is inhibited by the Ca^2+^-chelator, EGTA. This could indicate that Ca^2+^-calmodulin acts catalytically to load the DIII-DIV linker onto the CTD [19] (see below).

It had previously been suggested that Nav1.5 CTD EFLs could bind Ca^2+^ directly [7,9,13]. However, in Ca^2+^-binding EF hands, such as those occurring in calmodulin, the Ca^2+^-chelating acidic residues typically lie within turn loops between adjacent α-helices. This pattern is not seen in the CTD-EFL domain [10,20,21]. On the other hand, the CTD, with its significant homologies between Nav subtypes, illustrated for Nav1.4 and Nav1.5 (Figure 3A), binds calmodulin. So, this is the most likely mechanism by which the CTD senses [Ca^2+^]_i_. The IQ motif within helix 6 of the Nav1.5 CTD [22] (Figure 3A) can bind the apo-calmodulin C-lobe [14]. Additionally, both the EFL domain and the N-terminal of helix 6 can bind the apo-calmodulin N-lobe (Figure 3Ba). Following Ca^2+^-calmodulin binding, the IQ motif (Figure 3A) can bind the ‘semi-open state’ Ca^2+^-calmodulin C-lobe. But now a downstream, slightly overlapping N-lobe binding motif (NLBM) (Figure 3A) can bind a shifted Ca^2+^-calmodulin N-lobe (Figure 3Ba,b). An alternative structure (PDB: 6mud) for the Nav1.5 CTD Ca^2+^-calmodulin complex is shown in Figure 3Bc [23]. Here, the Ca^2+^-calmodulin N-lobe is untethered to the CTD, and the Ca^2+^-calmodulin C-lobe adopts a strikingly different orientation on helix 6 (Figure 3Bc). However, the CTD construct used in this structure contained a truncated NLBM motif, so that its binding to Ca^2+^-calmodulin N-lobe was likely compromised [23]. Interestingly, a BrS mutation A1924T [24] (Table 1) occurs within the Nav1.5 NLBM site, suggesting that the structure shown in Figure 3Bc could represent an abortive complex, leading to a BrS phenotype. Nav1.4 lacks a functioning NLBM (Figure 3A), whence this shift cannot occur (cf. [23] and it is striking that the rearrangements of calmodulin on the Nav1.4 CTD helix 6 are noticeably less pronounced compared with Nav1.5 (Figure 3Bd,e))

**Table 1:** Disease related C-terminal mutations in the Nav1.4 and Nav1.5 channel

Disease	Nav1.4 C-terminal associated mutations	Experimental results	References
Hyperkalaemic periodic paralysis	M1592V		(Rojas et al. [77])
Normokalaemic periodic paralysis	M1592V		(Xiuhai et al. [78])
Potassium-aggravated myotonia (Myo)	Q1633E		(Kubota et al. [79])
Paramyotonia Congenita (PMC)	F1705I		(Groome et al. [80])

Disease	Nav1.5 C-terminal associated mutations	Experimental results	References
Brugada Syndrome (BrS)	T1779M		(Kapplinger et al. [81, 89])
	E1784K		(Kapplinger et al. [81, 89])
	L1786EfsX2		(Kapplinger et al. [81])
	1795insD	Reduced peak I_Na._ ∼7 mV negative shift of steady-state inactivation and ∼8 mV positive shift of steady-state activation. Recovery from inactivation slowed	(Bezzina et al. [83], Kapplinger et al. [81])
	Y1795H	Accelerated onset of inactivation. Reduced peak I_Na._ Negatively shifted *V*_1/2_ of inactivation. Increased sustained I_Na._ Promoted entry to an intermediate or slowly developing inactivated state.	(Rivolta et al. [84])
	F1808IfsX3		(Kapplinger et al. [81])
	S1812X		(Kapplinger et al. [81])
	L1825P	Drug (e.g., cisapride) induced. Reduced peak I_Na_. Positively shifted *V*_1/2_ of activation. Negatively shifted *V*_1/2_ of inactivation.	(Makita et al. [85], Huang et al. [26])
	E1823HfsX10		(Kapplinger et al. [81])
	Q1832E		(Kapplinger et al. [81, 89])
	C1850S	Decreased I_Na_ density. ∼11 mV negative shift of *V*_1/2_ of inactivation	(Petitprez et al. [86])
	R1860KfsX13		(Kapplinger et al. [81])
	V1861I		(Kapplinger et al. [81])
	K1872N		(Kapplinger et al. [81, 89])
	S1904L	Enhanced late I_Na_ due to increased propensity of the Na^+^ channel to reopen during prolonged depolarization.	(Kapplinger et al. [81, 89])
	A1924T	∼9 mV negatively shifted *V*_1/2_ of steady-state activation.	(Rook et al. [24], Kapplinger et al. [81, 89])
	G1935S		(Kapplinger et al. [81, 89])
	E1938K		(Kapplinger et al. [81, 89])
	V1951L		(Priori et al. [82])
	I1968S	Decreased peak I_Na_	(Frustaci et al. [87])
	F2004L	Decreased peak and persistent I_Na_. Increased I_Na_ closed state inactivation. Accelerated slow inactivation accelerated and delayed recovery from inactivation.	(Bebarova et al. [88], Kapplinger et al. [89])
	F2004V		(Kapplinger et al. [81, 89])
	F2004dup		(Kapplinger et al. [81])
	V1777M		(Huang et al. [26], Kapplinger et al. [89])
Long QT Syndrome Type 3 (LQT3)	T1779M		(Huang et al. [26], Kapplinger et al. [89])
	E1784K		(Huang et al. [26], Kapplinger et al. [89])
	D1790G		(Huang et al. [26])
	Y1795C	Slowed onset of inactivation. Increased sustained I_Na_. Enhanced entry into an intermediate or slowly developing inactivated state	(Rivolta et al. [83], Kapplinger et al. [89], Huang et al. [26])
	1795insD	ECG: QT prolongation and ST segment elevation.	(Bezzina et al. [83], Huang et al. [26])
	D1819N		(Huang et al. [26])
	L1825P	Drug (e.g., cisapride) induced. Slowed I_Na_ decay and prominent TTX sensitive non-inactivating component.	(Makita et al. [85], Huang et al. [26])
	R1826H	Slowed I_Na_ decay and a 2–3 fold increase in late I_Na_.	(Ackerman et al. [90], Huang et al. [26])
	D1839G		(Huang et al. [26], Kapplinger et al. [89])
	H1849R	Slowed rate of steady-state inactivation. Prolonged action potential duration and delayed after depolarization.	(Musa et al. [91])
	R1897W		(Huang et al. [26], Kapplinger et al. [89])
	E1901Q		(Huang et al. [26], Kapplinger et al. [89])
	S1904L		(Bankston et al. [92], Kapplinger et al. [89], Huang et al. [26])
	Q1909R		(Huang et al. [26], Kapplinger et al. [89])
	R1913H		(Napolitano et al. [93])
	A1949S		(Tester et al. [94])
	V1951L		(Arnestad et al. [95], Kapplinger et al. [89])
	R1958Q		(Tester et al. [94], Kapplinger et al. [89])
	Y1977N		(Kapplinger et al. [81])
	F2004L	Increased persistent I_Na_	(Arnestad et al. [95], Kapplinger et al. [89])
	F2004V		(Kapplinger et al. [89])
	P2006A	Increased persistent I_Na_	(Kapplinger et al. [89], Amestad et al. [95])
	R2012C		(Kapplinger et al. [89])
Atrial fibrillation	R1826C		(Darbar et al. [96])
	V1951L		(Darbar et al. [96])
	V1951M		(Darbar et al. [96])
	N1986K		(Ellinor et al. [97])
	F2004L		(Darbar et al. [96])
Sick Sinus Syndrome (SSS)	D1792N		(Selly et al. [98])

The CTD and DIII-DIV linker of both Nav1.4 or Nav1.5 show mutations associated with specific disease phenotypes. These respectively involve skeletal or cardiac muscle electrophysiological function (Table 1) [25]. Interestingly, within the DIII-DIV linker, gain of Nav1.5 function LQT3 mutations cluster in Site A and affect residues that stabilize DIII-DIV linker binding to the intracellular face of DIV (Figure 2A,B) [26]. In the CTD, the LQT3 mutants tend to occur on helix 6, within and around the IQ motif anchoring apo-calmodulin, as well as contact sufaces between helix 6 and the EFL domain [26] (Figure 3A). These mutations are rescued by overexpressed calmodulin [27].

Contrastingly, loss of Nav1.5 function, Brugada Syndrome (BrS), mutations mainly occur in Site B of the DIII-DIV linker [26] (Figure 2A). One exception, however, is Site A residue Y1494. Mutations in this residue are associated with BrS, not LQT3 (Figure 2A). It may be significant that in the presumed inactivated state structure, residue Y1494 points away from the inactivation site on the intracellular region of DIV (Figure 2B), but in the Ca^2+^-calmodulin C-lobe/Site A complex, it now lies within the protein docking site (Figure 2C) [17]. Thus, BrS and LQT3-associated mutations in Site A, may perturb different molecular contacts. In the CTD, residues associated with BrS cluster particularly within the EFL cleft (Figure 3A,B). This could compromise the capture of the DIII-DIV linker and compromise recovery from inactivation (Figure 4A). In Nav1.4, mutations in two EFL residues, Q1633 and F1705 are associated with myotonia and paramyotonia congenita (PMC), respectively (Figure 3A). In the Nav1.4 EFL structure, these two residues lie suggestively close to each other, where they could help stabilize the EFL cleft (Figure 3Bd,e).

In summary: site A of the DIII-DIV linker can bind to an intracellular site on Nav α-subunit DIV, when the channel is in the inactivated state (Figure 2B). Yet it can also bind to the Ca^2+^-calmodulin C-lobe (Figure 2C) and to the CTD-EFL domain, when the channel is in the closed state [11]. Similarly, site B of the DIII-DIV linker can bind to DIV on the inactivated Nav α-subunit (Figure 2B), but also to the Ca^2+^-calmodulin N-lobe (Figure 2C). Furthermore, in several cases, the same amino acid residues contribute to the different binding states (Figure 2B,C). Thus, *within a given channel, these interaction states must be mutually exclusive*. Finally, as noted above, the cryo-EM structure (Figure 1B), suggests that the CTD does not bind the DIII-DIV linker when the channel is in the inactivated state [6]. The simplest interpretation is that these different binding states can only take place at specific points during the activation/inactivation/recovery from inactivation cycle of the channel and thus could help impose directionality onto the process.

This idea is outlined in schematic form for the whole Nav activity cycle in Figure 4A and for the role of calmodulin in the recovery from inactivation steps in Figure 4B. One may suggest that immediately after Nav1.5 inactivation, Site A and B, and the IFM motif of the DIII-DIV linker, are all fully engaged with their sites on the α-subunit DIII, and the CTD does not bind the DIII-DIV linker (Figure 4Ba). With an elevated [Ca^2+^]_i_, the interaction between Ca^2+^-calmodulin and the CTD is represented by structure PDB: 4jq0 (Figure 3Bb). As the membrane potential hyperpolarizes, the voltage sensing helices of DIII and DIV return to their resting states. Site A and the IFM motif detach from their sites on DIV (Figure 4Bb). The Ca^2+^-calmodulin C-lobe can then bind Site A, adopting the conformation shown in PDB: 4djc (Figure 2C, upper panel). Further rearrangements allow the Ca^2+^-calmodulin N-lobe to bind to Site B as in PDB: 5dbr (Figure 2C lower panel). Together, this could act like a ratchet to prevent the reattachment of Sites A and B and thus the IFM motif to DIV (Figure 4Bc) [18]. There must be further rearrangements to free the calmodulin C-lobe from Site A and the calmodulin N-lobe from site B, so that Site A can reattach to the cleft in the EFL domain of the CTD (Figure 4Bd–f) [21]. Since the affinity of calmodulin for Site A and B is strictly Ca^2+^-dependent, [18], this could take place as [Ca^2+^]_i_ returns to its resting state, (Figure 4Bf).

Other Nav sites may potentially be involved in Ca^2+^-mediated regulation. Thus, CaMKII-mediated phosphorylation of particular (Ser516, Ser571, and Thr594) residues within the DI-DII intracellular linker region increases late *I*_Na_ delaying action potential repolarization, characteristic of LQT3 [28]. However, an existence of calmodulin-KN93 interactions could result in attribution of modified protein function to CaMKII phosphorylation rather than calmodulin action. KN93 may also impair calmodulin-III–IV linker domain interaction and *I*_Na_ recovery from inactivation [29]. Phosphorylation at a protein kinase C specific site reduced peak *I*_Na_ and shifted (by −15 mV) steady state inactivation *V*_1/2_ [30]. Mutations at a Nav1.5 N-terminal domain calmodulin binding site down-regulated *I*_Na_ [31]. Elevated [Ca^2+^] may also up-regulate Nedd4-2 in turn targeting Nav1.5 for degradation via a CTD PY motif [32].

## *In vitro* cell expression systems exhibit Ca^2+^-dependent Na^+^ current modulation

The precise mechanisms of Ca^2+^-mediated channel modification amongst Nav isoforms are thus likely subjects of continued evaluation. Nevertheless, functional assessments confirm regulatory actions of Ca^2+^, Ca^2+^-calmodulin and apo-calmodulin on Nav1.4 and Nav1.5 electrophysiological properties. Table 2 summarizes available *in vitro* conventional patch-clamp explorations for Ca^2+^-dependent Nav1.4 and Nav1.5 current modulation variously employing heterologous tsA201, HEK293 and CHO expression systems. These quantified steady-state Na^+^ conductance (*g*_Na_) through its maximum currents, *I*_max_, and activation and/or inactivation half-maximal voltages, *V*_1/2_, and slope factors, *k*. Here, Nav1.4 and Nav1.5 are likely expressed in an absence of other accompanying *in vivo* proteins. Manoeuvres exploring alterations in [Ca^2+^]_i_ and calmodulin often used buffered, Ca^2+^-containing (0–10 µM), pipette solutions, to test for Ca^2+^, Ca^2+^-calmodulin or apo-calmodulin-mediated actions, on Nav1.4 and/or Nav1.5 C-terminal EF-hand or IQ domains, with some differences between reports [7,10,13,15,17,33–35].

**Table 2:** Ca^2+^ regulatory effects on Nav1.4 and Nav1.5 studied in heterologous expression systems

Experimental platform	Pipette buffer (mM concentrations unless otherwise stated)^1^		Shifts^2^ due to applied Ca^2+^				Shifts^2^ due to calmodulin (CaM)			
	0 [Ca^2+^]	X [Ca^2+^]	Activation		Inactivation		Activation		Inactivation	
			*I*_Na.max_	*V*_1/2_	*V*_1/2_	τ_fast_	*I*_Na.max_	*V*_1/2_	*V*_1/2_	τ_fast_
Nav1.5 (tsA201; Tan et al. [15])	10 EGTA	1 µM Ca^2+^ (1.0 EGTA/0.9 CaCl_2_)^3^	NIL	-	NIL	?Reduced	NIL	-	?Depol	Reduced
Nav1.4 (HEK293; Deschenes et al. [33])	10 BAPTA^4^		-	-	-	-	-	NIL^5^	NIL^5^	NIL^5^
	0 BAPTA	504 nM Ca^2+^ (3.7 CaCl_2_/5 BAPTA)^4^	-	NIL	?Depol	-	-	NIL	Hyper^6^	NIL
Nav1.5 (HEK293; Deschenes et al. [33])	0 BAPTA	504 nM Ca^2+^ (3.7 CaCl_2_/5 BAPTA)^4^	-	-	-	NIL	-	NIL	NIL	NIL
Nav1.5 (tsA201; Wingo et al. [7])	20 BAPTA	0–250 nM Ca^2+^ (0–13.4 CaCl_2_/20 BAPTA). 1 µM and 10 µM Ca^2+^ (0.9 CaCl_2_ or 1.0 CaCl_2_/1.0 BAPTA)^7^		NIL	Depol^8^	NIL	-	-	NIL	-
Nav1.4 (CHO-K1; Young and Caldwell [34])	5 EGTA		-	-	-	-	NIL	Hyper	Hyper^9^/NIL^10^	NIL
Nav1.4 (CHO-K1; Young and Caldwell [34])		10 µM Ca^2+^ (5 EGTA/4.9 CaCl_2_)^11^, ^12^	NIL	NIL	NIL	NIL	-	Hyper	NIL	NIL
Nav1.4 (HEK293; Young and Caldwell [34])	5 EGTA	10 µM Ca^2+^ (5 EGTA/ 4.9 CaCl_2_)	-	-	-	-	-	NIL	NIL	NIL
Nav1.5 (CHO-K1; Young and Caldwell [34])	5 EGTA	10 µM Ca^2+^ (5 EGTA/4.9 CaCl_2_)^13^	NIL	NIL	NIL	NIL	-	Hyper	NIL	NIL
Nav1.4 (tsA201; Shah et al. [13])	20 BAPTA	1 µM Ca^2+^ (1.0 BAPTA/0.9 CaCl_2_)	-	-	Depol^14^	-	-	-	-	-
Nav1.5 (HEK293; Biswas et al. [35])	20 BAPTA	10 µM Ca^2+^ (1.0 BAPTA/1.0 CaCl_2_) ^16^	NIL	NIL	Depol	Increased	NIL^15^	NIL^15^	Depol^15^	-
Nav1.5 (HEK293; Biswas et al. [35])		0.5 µM Ca^2+^ (5 BAPTA/ 4 CaCl_2_)^16^					NIL^15^	NIL^15^	NIL^15^	-
Nav1.5 (tsA201; Potet et al. [99])	20 BAPTA	10 µM Ca^2+^ (1.0 BAPTA/1.0 CaCl_2_)	-	-	Depol^17^	NIL				
Nav1.5 (tsA201; Chagot et al. [10])	20 BAPTA	1 µM Ca^2+^ (1.0 BAPTA/0.9 CaCl_2_).			Depol^18^					
Nav1.5 (tsA201; Sarhan et al. [17])	10 BAPTA	10 µM Ca^2+^ (1.0 BAPTA/1.0 CaCl_2_)	-	-	Depol^19^	NIL	-	-	-	-
Nav1.4 (HEK293; Ben-Johny et al. [36])	10 BAPTA	10 µM Ca^2+^ (10 HEDTA/5 CaCl_2_)	Reduced	-	NIL	-	-	-	-	-
Nav1.4 (HEK293; Ben-Johny et al. [36])	0.5 EGTA	Activation of co-expressed Cav2.1	Reduced	-	-	-	-	-	-	-
Nav1.4 (HEK293; Ben-Johny et al. [36])	Ca^2+^ uncaging; 1.0 citrate	0.5–2 µM Ca^2+^ (1.0 DMN/0.7 CaCl_2_) 2–8 µM Ca^2+^ (2 DMN/1.4 CaCl_2_)^21^	Reduced	-	NIL	-	Reduced^20^	-	-	-
Nav1.5 (HEK293; Ben-Johny et al. [36])	10 BAPTA	10 µM Ca^2+^ (10 HEDTA/5 CaCl_2_)	NIL	-	NIL	-	-	-	-	-
Nav1.5 (HEK293; Ben-Johny et al. [36])	0.5 EGTA	Activation of co-expressed Cav2.1	NIL	-	NIL					
Nav1.5 (HEK293; Ben-Johny et al. [36])	Ca^2+^ uncaging; 1.0 citrate	0.5–2 µM Ca^2+^ (1.0 DMN/0.7 CaCl_2_) 2–8 µM Ca^2+^ (2 DMN/1.4 CaCl_2_)	NIL	-	NIL					
Nav1.4 (*glt* skeletal muscle cells; Ben-Johny et al. [36])	Ca^2+^ uncaging; 1.0 citrate	0.5–2 µM Ca^2+^ (1.0 DMN/0.7 CaCl_2_) 2–8 µM Ca^2+^ (2 DMN/1.4 CaCl_2_)	Reduced	-	-	-	-	-	-	-
Nav1.5 (guinea-pig ventricular myocytes; Ben-Johny et al. [36])	Ca^2+^ uncaging; 1.0 citrate	0.5–2 µM Ca^2+^ (1.0 DMN/0.7 CaCl_2_) 2–8 µM Ca^2+^ (2 DMN/1.4 CaCl_2_)	NIL	-	-	-	-	-	-	-
Nav1.5 with Nav1.4 C-terminal (HEK293; Yoder et al. [38])	0.5 EGTA	Activation of co-expressed Cav2.1	Reduced^22^	-	-	-	Reduced	-	-	-
Nav1.5 with Nav1.4 C-terminal (HEK293; Yoder et al. [38])	Ca^2+^ uncaging; 1.0 citrate	0.5–2 µM Ca^2+^ (1.0 DMN/0.7 CaCl_2_) 2–8 µM Ca^2+^ (2 DMN/1.4 CaCl_2_)^23^	Reduced	-	NIL		Reduced			
Nav1.4 with Nav1.5 C-terminal (HEK293; Yoder et al. [38])	0.5 EGTA	Activation of co-expressed Cav2.1	NIL^24^	-	-	-	-	-	-	-
Nav1.4 with Nav1.5 C-terminal (HEK293; Yoder et al. [38])	Ca^2+^ uncaging; 1.0 citrate	0.5–2 µM Ca^2+^ (1.0 DMN/0.7 CaCl_2_) 2–8 µM Ca^2+^ (2 DMN/1.4 CaCl_2_)	NIL^24^	-	NIL					
Nav1.5 (rabbit ventricular myocytes; Casini et al. [40])	10 BAPTA	100 nM Ca^2+^ (CsCl/10 BAPTA)	NIL	NIL	NIL	NIL	-	-	-	-
Nav1.5 (rabbit ventricular myocytes; Casini et al. [40])		500 nM Ca^2+^ (CsCl/10 BAPTA)	Reduced	NIL	NIL	NIL	-	-	-	-
Nav1.5 (tsA201; Johnson et al. [18])	20 BAPTA	1.6 µM Ca^2+^ (5 HEDTA/0.9 Ca^2+^)							NIL	Increased^25^

1 ∼100 mM F^-^-containing pipette solutions except: Deschenes et al. [33] apart from C2C12 experiments (Sarhan et al. [17]; Ben-Johny et al. [36]; Yoder et al. [38]; Casini et al. [40]). DMN = DM Nitrophen.

2 Key: - = not studied; Nil = no effect; depol = depolarizing; hyper = hyperpolarizing shifts in *V*_1/2_;

3 Experiments performed with ±peptide 209–309 (antagonizing Ca^2+^-calmodulin-Nav1.5 binding), I1908E and L1912R IQ mutant and BrS mutant A1924T (Tan et al. [15]);

4 Pipette solution Cl^−^ or F^−^ and 0 Ca^2+^ (0 mM BAPTA) or 504 nM Ca^2+^ (3.7 mM Ca^2+^/5 mM BAPTA) gave similar results; further 10 µM KN92/KN93 and 100 nM CaMKII inhibitory autocamtide-2 (AIP) controls included;

5 Effects of 0 Ca^2+^ and of calmodulin-1234;

6 Double alanine IQ mutation hyperpolarized inactivation *V*_1/2_ and reduced decay constant relative to WT regardless of calmodulin mutation (Deschenes et al. [33]);

7 Experiments performed ± peptide 209–309 (antagonizing Ca^2+^-calmodulin-Nav1.5 binding;)

8 Depolarizing effect observed at >150 nM, saturated at 1 µM Ca^2+^, attenuated by EF hand D1790G LQT3 mutation, and abolished by 4× EF hand mutation (Wingo et al. [7]);

9 Effects of 0 Ca^2+^;

10 Effects of calmodulin-1234;

11 Experiments with 10 µM KN93/KN92, N- and C- terminal calmodulin mutants and Nav1.4/Nav1.5 C-terminal chimeras included;

12 IQ mutations I1727E and L1736R, showed unchanged channel properties relative to WT; I1727E blocked all effects of calmodulin and calmodulin-1234;

13 Experiments with 10 µM KN93/KN92, N- and C- terminal calmodulin mutants and Nav1.4/Nav1.5 C-terminal chimeras included (Young and Caldwell [34]);

14 Single, A1924T, but not double IQ mutation also caused depolarizing *V*_1/2_ shift (Shah et al. [13]);

15 Studies with calmodulin1234 included;

16 Ca^2+^ hyperpolarized inactivation *V*_1/2_ both in mutants lacking C-terminal and double alanine IQ mutation. Both EF hand LQT3 mutation D1790G and 4X mutation hyperpolarized inactivation *V*_1/2_ but were unresponsive to Ca^2+^ (Biswas et al. [35]);

17 A1924T mutant showed difference from WT only at 0 Ca^2+^ (Potet et al. [99]);

18 EF-2X mutation caused hyper and abolished Ca^2+^ action (Chagot et al. [10]);

19 No effect at 0.3 µM Ca^2+^ (Sarhan et al. [17]);

20 Time constants of Ca^2+^ dependent inactivation onset reported for different [Ca^2+^];

21 Double alanine IQ mutation caused Ca^2+^ dependent facilitation; myotonia mutants Q1626E and F1698I showed attenuated Ca^2+^-dependent inhibition and lesser reduction in I_max_ than WT. EF hand, D1621A and D1623A, mutations had no effect (Ben-Johny et al. [36]);

22 WT calmodulin and calmodulin-34 maintained Ca^2+^ dependent inactivation, calmodulin-12 resulted in loss of such inactivation.;

23 Nav1.5 mutant without the post IQ motif showed persistent Ca^2+^ dependent inhibition;

24 Ca^2+^ dependent inactivation persisted with Nav1.5 C-terminal domain lacking post IQ segment (Yoder et al. [38]);

25 Ca^2+^-calmodulin (but not apo-calmodulin) binding implicated in slowed kinetics of inactivation and accelerated recovery from inactivation, but not in Nav1.5 double mutants involving both sites A and B of II–III linker region.

However, their pipette [Ca^2+^] often significantly exceeded the Ca^2+^ dissociation constant, K_d_ of either the EGTA (67 nM) or 1,2-bis(2-aminophenoxy)ethane-N,N,N′,N′-tetra-acetic acid (BAPTA) (192 nM) pipette buffer, even as determined in the absence of Mg^2+^ [36]. Possible Ca^2+^-F^-^ binding (solubility product K_sp_ ∼ 3.45 × 10^−11^ M^3^) with use of (often ∼100 mM, giving [Ca^2+^] = 3.45 nM) CsF-containing pipette solutions to stabilize the whole-cell patch-clamp recordings, and intrinsic cell buffering properties, added additional uncertainties to detailed interpretation of their experimental results [37].

**Figure 5.**
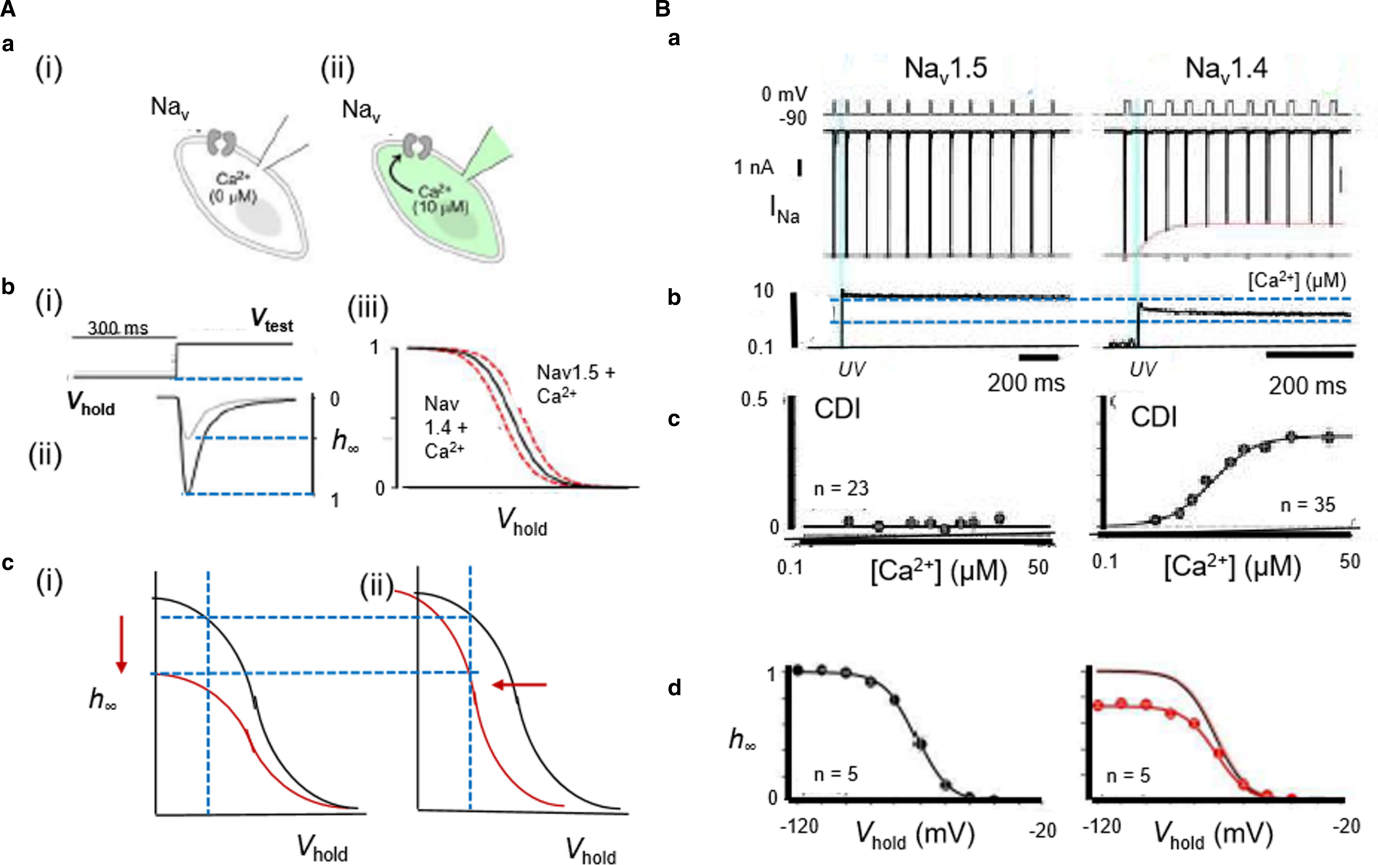
*In vitro* assessments of Ca^2+^-mediated Na^+^ current modulation in expression systems. (**A**) (**a**) Na^+^ channels characterized before (i) and following (ii) pipette dialysis with µM Ca^2+^. (**b**) Assessment of Ca^2+^ effects on Na^+^ current inactivation properties through (i) imposition of voltage steps from varying holding voltages, *V*_hold_, to a fixed test level, for measurement of (ii) corresponding Na^+^ currents and (iii) plotting fractional current remaining, h_∞_, at different V_hold_. (**c**) Alterations from normal (black) inactivation properties resulting in (i) reduction in maximum Na^+^ current or (ii) shift in the dependence of h_∞_ on V_hold_ (red). (B) Ca^2+^-dependent Na^+^ channel inhibition under Ca^2+^ photo-uncaging: (**a**) Na_V_1.5 peak currents unaffected but Na_V_1.4 peak currents decline with 10 µM Ca^2+^ uncaging. Gray dots, peak currents before (**b**) uncaging. (**c**) Ca^2+^-dependent inhibition plotted against Ca^2+^-step amplitude. (**d**) corresponding h_∞_ curves; upwardly scaled h_∞_ curve (red) similar to that obtained before uncaging (black). ((**A**)(**a**),(**b**) from Figure 1 and (**B**) from Figure 2 by permission (Ben-Johny et al. [36]).

Nevertheless, all these studies reported little or no effects on *k*. Nor did pipette Ca^2+^/EGTA, Ca^2+^/BAPTA or calmodulin manipulations alter *I*_max_. However, experiments instead buffering pipette Ca^2+^ using F^-^-free N-(2-hydroxyethyl)ethylenediamine-N,N′,N′-triacetic acid (HEDTA), and elevating [Ca^2+^]_i_ by Nitr-photo-uncaging, or activating co-expressed Cav1.2, contrastingly all reduced *I*_max_ in Nav1.4, or Nav1.5 chimeras expressing the Nav1.4 CTD (Figure 5). Contrastingly, they did not do so with Nav1.5 or Nav1.4 chimeras expressing the Nav1.5 CTD [36,38]. Inactivation *V*_1/2_s were unaffected and activation *V*_1/2_s not explored [36]. The remaining studies investigating *V*_1/2_ reported consistently unchanged activation *V*_1/2_s, but either altered or depolarized inactivation *V*_1/2_s, with no trends related to expression platform (Table 2). Nor did inactivation time constants alter, with two exceptions [15,18]. Finally, Ca^2+^ uncaging also revealed that FGF homologous factors (FHF) diminished Ca^2+^-calmodulin-regulation of Nav1.4 expressed in HEK293 cells, possibly involving allosteric sites within upstream CTD regions distinct from the calmodulin-binding interface [39].

Ca^2+^ uncaging investigations extending to skeletal myotubes derived from mouse *glt* cells similarly demonstrated Ca^2+^-mediated Nav1.4 regulation at sensitivities appropriate for physiological Ca^2+^ transients, but no such Nav1.5 regulation in adult guinea-pig ventricular myocytes [36]. However, in freshly isolated rabbit ventricular myocytes, [Ca^2+^]_i_ elevations produced by Ca^2+^-BAPTA (0–500 nM)-buffered patch-clamp electrode solutions or caffeine challenge caused parallel reductions in *I*_Na_ density, unit channel amplitudes and maximum action potential upstroke rates (d*V*/d*t*)_max_, without altering steady state voltage dependences of *I*_Na_ activation or inactivation [40]. Cultured rat neonatal ventricular cardiomyocytes also showed altered Nav expression with more sustained alterations in intracellular Ca^2+^ homeostasis. Nav1.5 mRNA levels then altered in parallel with decreases or increases in whole cell patch-clamp *I*_Na_ with 24 h sustained elevations (10 mM) or BAPTA-AM-mediated reductions of culture media [Ca^2+^]. These also occurred without alterations in single conductance, or activation and inactivation properties [41].

These varied observations could arise from a range of possible Nav Ca^2+^ sensing mechanisms, including direct Ca^2+^ binding to the first EF-like hand [7,15,35], or Ca^2+^-calmodulin or apo-calmodulin binding to, the CTD [34,36]. The latter possibilities were compatible with reported calmodulin binding to peptide channel fragments [42,43]. Finally, structural studies invoked possible Nav regulatory sites alternative to the CTD including the III–IV loop [17]. At all events, this available evidence permits a direct *in vivo* regulation of Nav-mediated excitable activity by intracellular Ca^2+^, involving mechanisms highly conserved among all nine Nav isoforms. This could complement or replace hypotheses invoking [Ca^2+^]_i_-mediated increases in electrogenic Na^+^/Ca^2+^ exchanger (NCX) activity in cardiac muscle under pro-arrhythmic conditions [44]. The latter may mediate delayed after depolarization (DAD) phenomena and is also implicated in altering action potential recovery as opposed to initiation and propagation activity. NCX may also increase [Na^+^]_i_ thereby influencing transmembrane Na^+^ electrochemical gradients. However, this would involve µM-levels corresponding to the altered [Ca^2+^]_i_ as opposed to normal background nM-[Na^+^]_i_ levels. Furthermore, NCX activity is not a prominent normal skeletal as opposed to cardiac muscle feature. Nevertheless, in either event, over the long term, reduced or increased background [Ca^2+^]_i_ resulting from sustained low or high firing rates could furnish a form of Ca^2+^ memory modifying Nav expression or gating and therefore its availability for driving action potential upstroke and propagation. In skeletal muscle, this could reduce cell excitability permitting recovery from fatiguing stimulation. However, the accompanying conduction velocity (CV) reductions could contribute to pathological cardiac arrhythmic or epileptiform nerve cell phenotypes.

## Native skeletal and cardiac myocytes show acute Ca^2+^-dependent *I*_Na_ modulation

**Figure 6.**
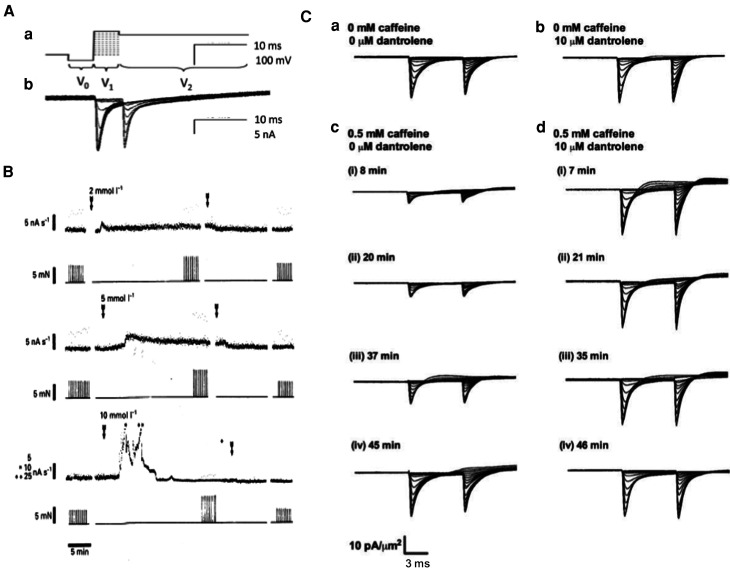
Na^+^ current down-regulation in native murine skeletal muscle fibres by altered Ca^2+^ homeostasis following caffeine induced ryanodine receptor (RyR) activation, abrogated by dantrolene mediated RyR antagonism. (**A**)(**a**) Double pulse protocol from a hyperpolarized prepulse potential V_0_ to activating voltage V_1_ followed by further depolarization to fixed depolarized voltage V_2_, respectively assessing (**b**) Na^+^ current activation and subsequent inactivation produced by the voltage step to V_1_. (**B**) 2–10 mM caffeine increases integrated background aequorin Ca^2+^ signal (upper trace) and twitch force (lower trace) in rat fast twitch muscle at 25°C over timecourses dependent upon caffeine concentration. Arrows denote periods of caffeine exposure. (**C**) Families of loose-patch clamp membrane currents in response to the double pulse protocol before (**a**, **b**) and at successive intervals ((i)-(iv)) following introduction (**c**, **d**) of caffeine (0.5 mM) before (**a**, **c**) and following (**b**, **d**) addition of dantrolene (10 µM). Currents expressed as current densities (pA/µm^2^) through 28–32 µm pipette diameters.((**A**) from Figure 2 by permission (Fryer & Neering [47]); (**B**) from Figure 3 by permission (Sarbjit-Singh et al. [48]).

*In vivo* Ca^2+^-dependent Nav modulation was observed in native cardiac or skeletal myocytes in intact physiological systems and clinical disease models. Use of loose, as opposed to conventional cell-attached, patch-clamp methods, avoided Ca^2+^ perturbations produced by the measurement method itself. *I*_Na_ families recorded from voltage steps from resting to sequentially depolarized activating test potentials, followed by further pulses to a fixed depolarized level to evaluate the resulting channel inactivation (Figure 6Aa,b) were compared before and following perturbations of their *in vivo* Ca^2+^ homeostatic mechanisms. Studies in both skeletal and cardiac myocytes demonstrated potentially physiologically significant negative feedback regulation of Nav1.4 and Nav1.5 by RyR-mediated release of intracellularly stored SR Ca^2+^. In murine skeletal muscle, acute RyR2 activation by the exchange protein directly activated by cAMP (Epac) by the activator 8-(4-chlorophenylthio)adenosine-3′,5′-cyclic monophosphate (8-CPT, 1 µM) [45], reduced maximum *I*_Na_ whilst leaving *V*_1/2_ values unchanged, actions abrogated by the RyR-inhibitor dantrolene (10 µM) [46]. The RyR agonist caffeine, at concentrations of 0.5 or 2 mM, produced sustained activation or transient activation followed by inactivation, of RyR-mediated SR Ca^2+^ release and corresponding parallel alterations in [Ca^2+^]_i_ [47; Figure 6B]. These changes directly paralleled time-dependent decreases or increases in peak *I*_Na_ values (Figure 6Cc,d) also abrogated by dantrolene (10 µM). Finally, dantrolene applied by itself produced small increases in *I*_Na_, suggesting inhibitory effects of even background Ca^2+^ release on *I*_Na_ ((Figure 6Ca,b) [48], potentially through formation of microdomains localizing [Ca^2+^]_i_ heterogeneities in junctional regions separating the T-tubular and SR membranes [49]

**Figure 7.**
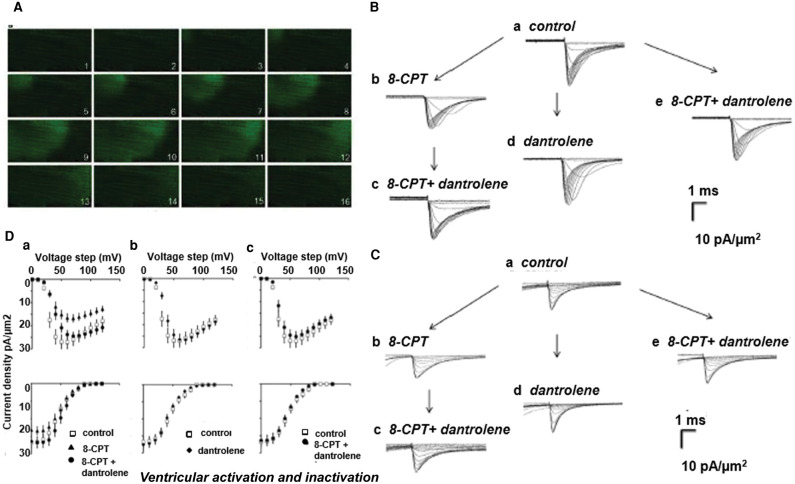
Na^+^ current reduction in native murine cardiomyocytes by altered Ca^2+^ homeostasis following ryanodine receptor (RyR) activation by the Epac activator 8-CPT, abrogated by dantrolene mediated RyR antagonism. (**A**) Epac-induced wave of elevated cytosolic [Ca^2+^] ([Ca^2+^]_i_) shown in 41.0 × 20.5 µm confocal microscope fluo-3 images taken in successive 65 ms intervals within isolated ventricular myocyte. (**B**, **C**) Families of loose-patch clamp ionic current densities in a ventricular preparation; pulse protocol investigating Na^+^ channel activation and inactivation as in Figure 5A. Na^+^ currents in response to (**B**) activation by depolarization to level V_1_ and (**C**) following their inactivation, to final level V_2_ following their inactivation at level V_1_. Recordings made (**a**) before pharmacological challenge, (**b**) in the presence of 8-CPT (1 µM) alone or (**c**) following further addition of dantrolene, (**d**) after adding dantrolene alone or (**e**) combined with 8-CPT. (**D**) Corresponding dependences of Na^+^ current activation (top row) and inactivation (bottom row) (mean ± SEM) upon voltage V_1_ (**a**) before (open squares) and following introduction of 8-CPT (filled triangles) and 8-CPT and dantrolene combined (filled circles), (**b**) before (open squares) and following introduction of dantrolene (filled diamonds), (**c**) before (open squares) and following introduction of a combination of 8-CPT and dantrolene (filled circles).((**A**) From Figure 8 by permission (Hothi et al. [45]); (**B**), (**C**) from Figure 2 and (**D**) from Figure 4 by permission (Valli et al. [76]).

Elevating [Ca^2+^]_i_ by applications of high extracellular [Ca^2+^], caffeine, and the SR Ca^2+^ ATPase inhibitor cyclopiazonic acid in murine atria [50], in addition to 8-CPT in murine atria and ventricles, all reduced mean peak inward *I*_Na_. 8-CPT (1 µM) induced Ca^2+^ homeostatic changes manifesting as spectrofluometrically measured spontaneous Ca^2+^ waves in murine atrial myocytes (Figure 7A) [45]. These findings accompanied 30–50% reductions in inward *I*_Na_ (Figure 7B,C), abrogated by dantrolene (10 µM), which by itself left *I*_Na_ at pre-treatment levels. Inactivation *V*_1/2_ and *k* (Figure 7D), and time constants for Na^+^ current recovery from inactivation remained unchanged [51]. Intracellular sharp microelectrode membrane potential recordings in intact Langendorff-perfused preparations correspondingly demonstrated reduced maximum atrial and ventricular (d*V*/d*t*)_max_ [51]. Action potential latencies reflecting delayed conduction increased while action potential durations and refractory periods were unchanged. The hearts also showed increased ventricular arrhythmic incidences following rapid pacing or extrasystolic stimuli [52].

## Ca^2+^-dependent *I*_Na_ modulation may underly skeletal muscle cold-aggravated myotonia

A first clinical example of a C-terminal Nav1.4, *SCN4A*, mutation associated with human disease is cold-aggravated myotonia, which causes transient myotonic stiffness or renders fibres transiently inexcitable resulting in a periodic paralysis (Table 1). The SCN4A mutant concerned contained two predicted amino acid substitutions, a DIS5-S6 loop T323M and an intracellular C-terminus F1705I substitution. Whole cell patch clamp *I*_Na_ from transiently transfected HEK293 cells expressing Nav1.4-T323M were indistinguishable from WT, consistent with a benign polymorphism. However, Nav1.4-F1705I channels showed a slowed fast inactivation with a positive 8.6 mV shift in steady-state voltage-dependence often associated with myotonia, but normal activation, recovery from fast inactivation or persistent current [53].

## Ca^2+^-dependent *I*_Na_ modulation may mediate pro-arrhythmic phenotypes in a catecholaminergic polymorphic ventricular tachycardia model

The hereditary pro-arrhythmic condition catecholaminergic polymorphic ventricular tachycardia (CPVT), is associated with gene mutations involving ryanodine receptor type 2 (*RYR2*), calsequestrin (*CASQ2*), triadin (*TRDN*) or calmodulin (*CALM1*, *CALM2* and *CALM3*) [54]. It clinically presents as potentially fatal bidirectional, and mono and polymorphic ventricular tachycardia (VT) provoked by adrenergic stress. Experimental murine *RyR2-P2328S* ventricles showed abnormal RyR2-mediated diastolic [Ca^2+^]_i_ elevations [55]. Homozygotic murine *RyR2-P2328S* ventricles showed reduced loose patch-clamp *I*_Na_ and possible additional evidence for down-regulated Nav1.5 expression [56]. Intrinsically beating murine *RyR2-P2328S* hearts recapitulated the clinical pro-arrhythmic phenotypes on isoproteronol and caffeine challenge. Intracellular floating microelectrode and multi-electrode array recordings then demonstrated correspondingly reduced (d*V*/d*t*)_max_, and ventricular epicardial CVs, particularly in homo- as opposed to heterozygotic, *RyR2-P2328S/+,* hearts, changes not observed in wild-type (WT) controls [57].

**Figure 8.**
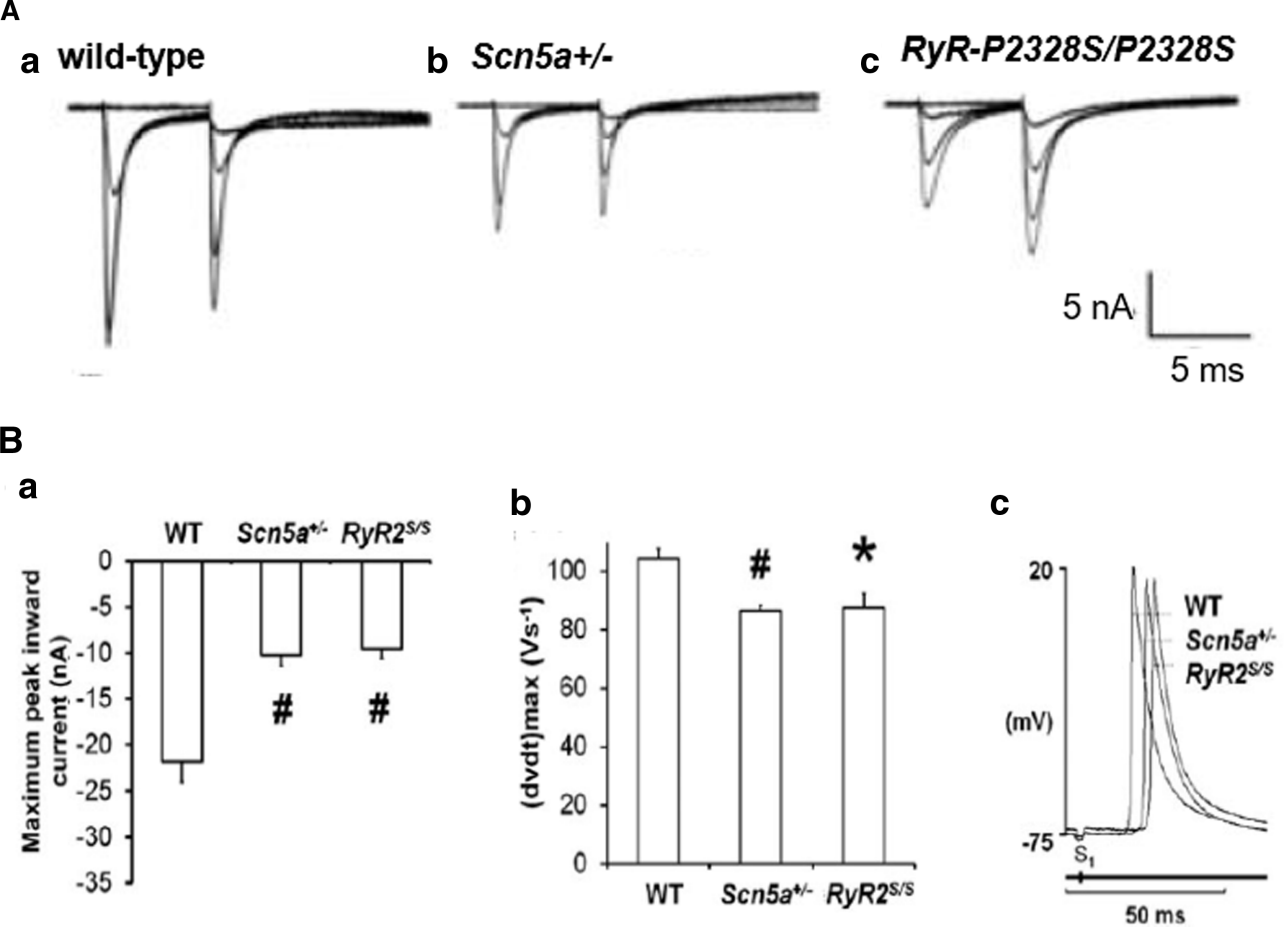
Altered Na^+^ current function paralleling Na^+^ channelopathy occurs in a murine pro-arrhythmic catecholaminergic polymorphic ventricular tachycardia model. (**A**) Loose-patch membrane current recordings in (**a**) WT, (**b**) *Scn5a*+/− and (**c**) *RyR2*-P2328S/P2328S atria. (**B**)(**a**) The resulting maximum peak inward currents (# *P* < 0.005). (**b**) Maximum upstroke rates ((d*V*/d*t*)_max_) and (**c**) waveforms showing conduction delays in left atrial intracellular action potentials. (Adapted from Figure 5 by permission (King et al. [50]).

CPVT is also associated with atrial fibrillation similarly attributed to abnormal Ca^2+^ homeostasis particularly following increased sympathetic tone [58]. In superfused *RyR2-P2328S/P2328S* atrial preparations, loose patch clamp measurements also demonstrated reduced peak *I*_Na_ with otherwise normal activation and inactivation current–voltage relationships (Figure 8A,Ba) [50]. Floating intracellular microelectrode measurements demonstrated reduced (d*V*/d*t*)_max_ and interatrial CVs though normal action potential duration amplitudes and refractory periods (Figure 8Bb,c) while multi-electrode arrays detected reduced atrial epicardial action potential CVs in *RyR2-P2328S/P2328S* atria when compared with WT [59]. Intrinsically active and regularly stimulated *RyR2-P2328S/P2328S* but not wild-type atria correspondingly showed frequent sustained tachyarrhythmias, delayed afterdepolarizations and ectopic action potentials. Extrasystolic S2 stimulation provoked arrhythmia at longer S1S2 intervals in *RyR2-P2328S/P2328S* than WT atria, nevertheless corresponding to similar (d*V*/d*t*)_max_, and effective interatrial CVs as in WT [59]. Gain-of-function skeletal muscle *RYR1* mutations are associated with a malignant hyperthermia typically following halothane anaesthesia. Reports of increased slowly inactivating inward, tetrodotoxin sensitive current in cultured human malignant hyperthermia skeletal myocytes may prompt further investigations into possible electrophysiological, Nav1.4 phenotypes [60].

## Anti-arrhythmic targeting of Ca^2+^ homeostasis in clinical CPVT, cardiac failure and hypertrophic cardiomyopathies

**Figure 9.**
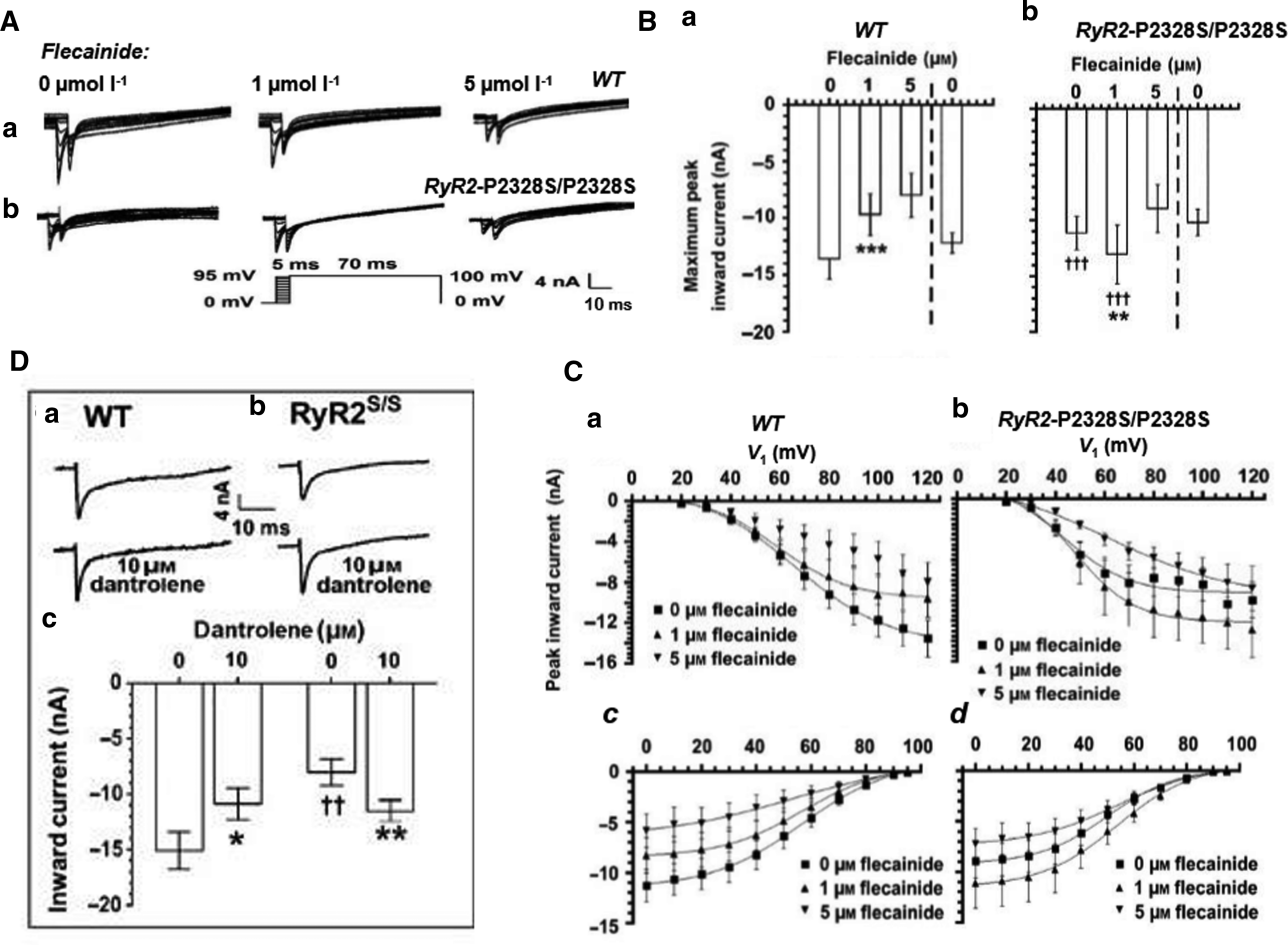
Ca^2+^ sensitivity of Nav1.5 accounts for paradoxical effects on Na^+^ currents of low dose flecainide used in clinical CPVT monotherapy. Comparisons of murine (**a**) WT and (**b**) *RyR2*-P2328S/P2328S left atria in the presence of 0, 1 and 5 µM flecainide showing: (**A**) Paradoxical actions of progressively increasing flecainide concentrations on Na^+^ current activation and inactivation properties in response to families of depolarizing activating steps each succeeded by a step to a constant 95 mV depolarization. (**B**) (**a**, **b**) Maximum peak currents with exposure followed by withdrawal of flecainide. (**C**) Activation (**a**,**b**) and inactivation (**c**,**d**) current–voltage relationships and their fits to Boltzmann functions in WT (**a**,**c**) and *RyR2*-P2328S/P2328S (**b**,**d**). (**D**) Similar paradoxical effects shown by membrane currents in response to an 80 mV depolarizing step before and following challenge by the RyR blocker dantrolene (10 µM). ((**A**), (**C**)(**c**,**d**) from Figure 4 and (**B**), (**C**)(**a**, **b**) and (**D**) from Figure 3 by permission (Salvage et al. [67]).

The above properties may underpin reported paradoxical pro- and anti-arrhythmic actions of low (1 µM) flecainide concentrations in WT and *RyR2-P2328S/P2328S* murine atria. Flecainide blocks both Nav1.5 and RyR2 with IC_50_s of 2–7 µM and 5–11 µM, respectively [61–63]. Either effect could potentially rescue an elevated [Ca^2+^]_i_. On the one hand, flecainide's Class Ic Nav1.5 blocking action causes a pro-arrhythmic CV slowing; however, action of a consequently reduced [Na^+^]_i_ on NCX could reduce pro-arrhythmic [Ca^2+^]_i_ elevations [64–66]. In intact WT hearts, flecainide (1 µM) exerted atrial pro-arrhythmic effects, accompanying reduced loose patch clamp *I*_Na_ and multi-electrode array recorded CV, whilst sparing refractory periods (Figure 9Aa,Ba,Ca). On the other hand, in *RyR2-P2328S/P2328S* atria, flecainide paradoxically rescued increased arrhythmic frequency. However, in contrast with its Nav1.5 inhibitory action in WT, it rescued *I*_Na_ and maintained CV at WT values, leaving refractory periods unchanged (Figure 9Ab,Bb,Cb), effects directly replicated by the RyR blocker dantrolene (Figure 9D) [67]. These findings together suggested a rescue of the arrhythmic phenotype by RyR2 block causing Nav1.5 rescue rather than Nav1.5 block. RyR2 inhibition would reduce the elevated diastolic Ca^2+^ and its pro-arrhythmic inhibition of Nav1.5 [67]. The latter mechanism of action could underlie anti-arrhythmic effects of monotherapeutic low-dose flecainide introduced to treat clinical CPVT [62,68–71].

Ca^2+^-mediated regulation of Nav1.5 may also contribute to commoner pro-arrhythmic cardiac conditions associated with spontaneous SR Ca^2+^ leak. The latter was reported in peroxisome proliferator activated receptor-γ coactivator-1 (PGC-1) transcriptional coactivator deficient (*Pgc1*-β^−/−^) murine models for pro-arrhythmic metabolic changes related to ageing, obesity and diabetes mellitus [72]. Atrial fibrillation, cardiac failure and hypertrophic cardiomyopathies are also accompanied by spontaneous SR Ca^2+^ leak. Classically, SR Ca^2+^ leak is implicated in a pro-arrhythmic activation of inward depolarizing, NCX current [44]. However, the pro-arrhythmic phenotypes in *Pgc1*-β^−/−^ atria and ventricles were also associated with reduced *I*_Na_ [73,74], (d*V*/d*t*)_max_ and CVs [75,76]. A decreased *I*_Na_ in these experimental conditions as well as in clinical heart failure or atrial fibrillation slowing action potential CV could contribute pro-arrhythmic substrate.

## Perspectives

- Action potential generation by Na^+^ channel (Nav) activation and the resulting release of intracellular Ca^2+^ stores underly skeletal and cardiac myocyte excitation-contraction coupling abnormalities which underly a wide range of human genetic diseases.
- Nav channels possess sites directly or indirectly binding Ca^2+^ potentially of regulatory importance in their reciprocal Ca^2+^-mediated feedback regulation. Evidence from cell expression systems, native myocytes and normal and disease models demonstrate such Ca^2+^-mediated Nav regulation effects.
- Future studies may correlate this molecular evidence bearing particularly on the Nav C-terminal and III–IV linker domains and biophysical studies of Na^+^ channel function with associated clinical conditions.

## Open Access Statement

Open access for this article was enabled by the participation of University of Cambridge in an all-inclusive Read & Publish pilot with Portland Press and the Biochemical Society under a transformative agreement with JISC.

## Abbreviations

BAPTA: bis(2-aminophenoxy)ethane-N,N,N′,N′-tetra-acetic acid
CPVT: catecholaminergic polymorphic ventricular tachycardia
CTD: C-terminal domains
CV: conduction velocity
EFL: EF-like hand
NCX: Na^+^/Ca^2+^ exchanger
NLBM: N-lobe binding motif
PMC: paramyotonia congenita
SR: sarcoplasmic reticular
